# (2*Z*)-2-{[*N*-(2-Formyl­phen­yl)-4-methyl­benzene­sulfonamido]­meth­yl}-3-(4-methyl­phen­yl)prop-2-ene­nitrile

**DOI:** 10.1107/S1600536812010628

**Published:** 2012-03-17

**Authors:** D. Kannan, M. Bakthadoss, R. Madhanraj, S. Murugavel

**Affiliations:** a Department of Organic Chemistry, University of Madras, Maraimalai Campus, Chennai 600 025, India; b Department of Physics, Ranipettai Engineering College, Thenkadappathangal, Walaja 632 513, India; c Department of Physics, Thanthai Periyar Government Institute of Technology, Vellore 632 002, India

## Abstract

In the title compound, C_25_H_22_N_2_O_3_S, the sulfonyl-bound benzene ring forms dihedral angles of 36.8 (2) and 81.4 (2)°, respectively, with the formyl­benzene and methyl­benzene rings. The mol­ecular conformation is stabilized by an intra­molecular C—H⋯O hydrogen bond, which generates an *S*(5) ring motif. The crystal packing is stabilized by C—H⋯O hydrogen bonds, which generate *C*(11) chains along the *b* axis. The crystal packing is further stabilized by π–π inter­actions [centroid–centroid distance = 3.927 (2) Å].

## Related literature

For background to the pharmacological uses of sulfonamides, see: Korolkovas (1988 ▶); Mandell & Sande (1992 ▶). For related structures, see: Madhanraj *et al.* (2012 ▶); Aziz-ur-Rehman *et al.* (2010 ▶). For hydrogen-bond motifs, see: Bernstein *et al.* (1995 ▶).
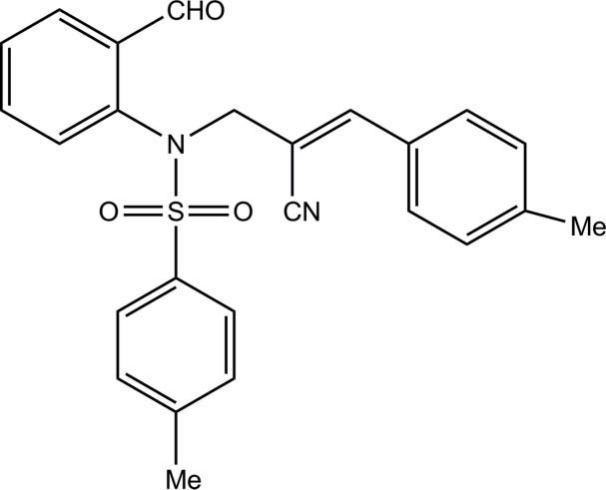


## Experimental

### 

#### Crystal data

C_25_H_22_N_2_O_3_S
*M* *_r_* = 430.51
Orthorhombic, 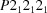

*a* = 8.9432 (5) Å
*b* = 10.3004 (6) Å
*c* = 24.9240 (15) Å
*V* = 2296.0 (2) Å^3^
*Z* = 4
Mo *K*α radiation
μ = 0.17 mm^−1^
*T* = 293 K
0.25 × 0.23 × 0.17 mm

#### Data collection

Bruker APEXII CCD diffractometer
Absorption correction: multi-scan (*SADABS*; Sheldrick, 1996 ▶) *T*_min_ = 0.959, *T*_max_ = 0.972
12317 measured reflections
4663 independent reflections
3385 reflections with *I* > 2σ(*I*)
*R*_int_ = 0.024

#### Refinement

*R*[*F* ^2^ > 2σ(*F* ^2^)] = 0.043
*wR*(*F* ^2^) = 0.117
*S* = 1.02
4663 reflections
282 parameters
H-atom parameters constrained
Δρ_max_ = 0.19 e Å^−3^
Δρ_min_ = −0.23 e Å^−3^
Absolute structure: Flack (1983 ▶), 1990 Friedel pairs
Flack parameter: 0.19 (9)

### 

Data collection: *APEX2* (Bruker, 2004 ▶); cell refinement: *APEX2* and *SAINT* (Bruker, 2004 ▶); data reduction: *SAINT* and *XPREP* (Bruker, 2004 ▶); program(s) used to solve structure: *SHELXS97* (Sheldrick, 2008 ▶); program(s) used to refine structure: *SHELXL97* (Sheldrick, 2008 ▶); molecular graphics: *ORTEP-3* (Farrugia (1997 ▶); software used to prepare material for publication: *SHELXL97* and *PLATON* (Spek, 2009 ▶).

## Supplementary Material

Crystal structure: contains datablock(s) global, I. DOI: 10.1107/S1600536812010628/tk5066sup1.cif

Structure factors: contains datablock(s) I. DOI: 10.1107/S1600536812010628/tk5066Isup2.hkl

Supplementary material file. DOI: 10.1107/S1600536812010628/tk5066Isup3.cml

Additional supplementary materials:  crystallographic information; 3D view; checkCIF report

## Figures and Tables

**Table 1:** Hydrogen-bond geometry (Å, °)

*D*—H⋯*A*	*D*—H	H⋯*A*	*D*⋯*A*	*D*—H⋯*A*
C15—H15*A*⋯O3	0.97	2.45	2.904 (3)	109
C23—H23⋯O1^i^	0.93	2.50	3.142 (4)	127

Symmetry code: (i) 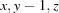.

**Fig. 1.**
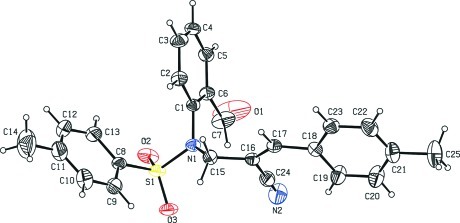
The molecular structure of the title compound with the atom numbering scheme. Displacement ellipsoids are drawn at the 20% probability level. H atoms are presented as a small cycles of arbitrary radius.

**Fig. 2.**
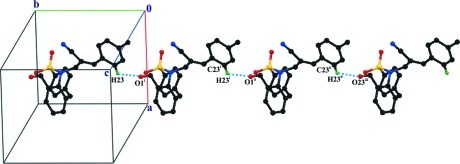
Part of the crystal structure of (I) showing C—H···O hydrogen bonds (dotted lines), with the formation of C(11) chains along b axis. [Symmetry codes: (i)x, -1 + y, z; (ii)x, -2 + y, z; (iii)x, -3 + y, z].

**Fig. 3.**
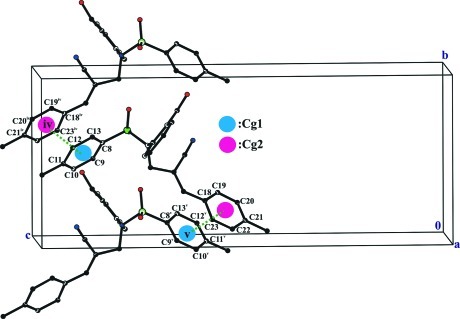
A view of the π—π interactions (dotted lines) in the crystal structure of the title compound. Cg1 and Cg2 denotes centroids of the C8–C13 benzene ring and C18–C23 benzene ring, respectively. [Symmetry codes: (iv)-x, 1/2 + y, 3/2 - z; (v)-x, -1/2 + y, 3/2 - z].

## supplementary crystallographic information

### Comment

Sulfonamide drugs are widely used for the treatment of certain infections caused by Gram-positive and Gram-negative microorganisms, some fungi, and certain protozoa (Korolkovas, 1988; Mandell & Sande, 1992). In view of this biological importance, the crystal structure of the title compound (I) has been determined and the results are presented here.

Fig. 1. shows a displacement ellipsoid plot of (I), with the atom numbering scheme. The S1 atom shows a distorted tetrahedral geometry, with the O2—S1—O3 [119.9 (1)°] and N1—S1—C8[107.5 (1)°] angles deviating from ideal tetrahedral values. The sum of bond angles around N1 (351.9°) indicates that N1 has *sp*^2^ hybridization. The sulfonyl bound benzene (C8–C13) ring forms dihedral angles of 36.8 (2) and 81.4 (2)°, respectively, with the formyl benzene (C1–C6) and methylbenzene (C18—C23) rings. The dihedral angle between formyl benzene and methylbenzene rings is 87.4 (1)°. The carbonitrile side chain (C16–C24–N2) is almost linear, with the angle around the C24 atom being 177.1 (3)°. The geometric parameters of the title molecule agrees well with those reported for similar structures (Madhanraj *et al.*, 2012, Aziz-ur-Rehman *et al.*, 2010).

The molecular structure is stabilized by a C15—H15A···O3 intramolecular hydrogen bond, forming a *S*(5) ring motif (Bernstein *et al.*, 1995) (Table 1). The crystal packing is stabilized by intermolecular C—H···O hydrogen bonds. Atom C23 in the molecule at (*x, y, z*) donates one proton to atom O1 at (*x,* -1 *+ y, z*), forming a C(11) chain along the *b* axis (Fig. 2). The crystal packing is further stabilized by π—π interactions with centroid—centroid distances: *Cg*1—*Cg*2^iv^ = 3.927 (2) Å and *Cg*2—*Cg*1^v^ = 3.927 (2) Å (Fig. 3; *Cg*1 and *Cg*2 are the centroids of C8–C13 benzene ring and C18–C23 benzene rings, respectively, symmetry code as in Fig. 3).

### Experimental

A solution of *N*-(formylphenyl)(4-methylbenzene)sulfonamide (1 mmol, 0.275 g) and potassium carbonate (1.5 mmol, 0.207 g) in acetonitrile was stirred for 15 minutes at room temperature. To this solution, (*E*)-2-(bromomethyl)-3-(4-methylphenyl)prop-2-enenitrile (1.2 mmol, 0.283 g) was added drop wise until the addition was completed. After the completion of the reaction, as indicated by TLC, acetonitrile was evaporated off. Ethylacetate (15 ml) and water (15 ml) were added to the crude mass. The organic layer was dried over anhydrous sodium sulfate. Removal of solvent led to the crude product, which was purified through a pad of silica gel (100–200 mesh) using ethylacetate and hexanes (1:9) as solvents. The pure title compound was obtained as a colourless solid (0.41 g, 95% yield). Recrystallization was carried out using ethylacetate as solvent.

### Refinement

H atoms were positioned geometrically, with C—H = 0.93–0.98 Å and constrained to ride on their parent atom with *U*_iso_(H)=1.5*U*_eq_ for methyl H atoms and 1.2*U*_eq_(C) for other H atoms.

### Crystal data

**Table:**

C_25_H_22_N_2_O_3_S	*F*(000) = 904
*M**_r_* = 430.51	*D*_x_ = 1.245 Mg m^−^^3^
Orthorhombic, *P*2_1_2_1_2_1_	Mo *K*α radiation, λ = 0.71073 Å
Hall symbol: P 2ac 2ab	Cell parameters from 4690 reflections
*a* = 8.9432 (5) Å	θ = 2.1–26.4°
*b* = 10.3004 (6) Å	µ = 0.17 mm^−^^1^
*c* = 24.9240 (15) Å	*T* = 293 K
*V* = 2296.0 (2) Å^3^	Block, colourless
*Z* = 4	0.25 × 0.23 × 0.17 mm

### Data collection

**Table:**

Bruker APEXII CCD diffractometer	4663 independent reflections
Radiation source: fine-focus sealed tube	3385 reflections with *I* > 2σ(*I*)
Graphite monochromator	*R*_int_ = 0.024
Detector resolution: 10.0 pixels mm^-1^	θ_max_ = 26.4°, θ_min_ = 2.1°
ω scans	*h* = −11→8
Absorption correction: multi-scan (*SADABS*; Sheldrick, 1996)	*k* = −12→9
*T*_min_ = 0.959, *T*_max_ = 0.972	*l* = −30→31
12317 measured reflections	

### Refinement

**Table:**

Refinement on *F*^2^	Secondary atom site location: difference Fourier map
Least-squares matrix: full	Hydrogen site location: inferred from neighbouring sites
*R*[*F*^2^ > 2σ(*F*^2^)] = 0.043	H-atom parameters constrained
*wR*(*F*^2^) = 0.117	*w* = 1/[σ^2^(*F*_o_^2^) + (0.065*P*)^2^ + 0.0718*P*] where *P* = (*F*_o_^2^ + 2*F*_c_^2^)/3
*S* = 1.02	(Δ/σ)_max_ = 0.001
4663 reflections	Δρ_max_ = 0.19 e Å^−^^3^
282 parameters	Δρ_min_ = −0.23 e Å^−^^3^
0 restraints	Absolute structure: Flack (1983), 1990 Friedel pairs
Primary atom site location: structure-invariant direct methods	Flack parameter: 0.19 (9)

### Special details

**Table:**

Geometry. All e.s.d.'s (except the e.s.d. in the dihedral angle between two l.s. planes) are estimated using the full covariance matrix. The cell e.s.d.'s are taken into account individually in the estimation of e.s.d.'s in distances, angles and torsion angles; correlations between e.s.d.'s in cell parameters are only used when they are defined by crystal symmetry. An approximate (isotropic) treatment of cell e.s.d.'s is used for estimating e.s.d.'s involving l.s. planes.
Refinement. Refinement of *F*^2^ against ALL reflections. The weighted *R*-factor *wR* and goodness of fit *S* are based on *F*^2^, conventional *R*-factors *R* are based on *F*, with *F* set to zero for negative *F*^2^. The threshold expression of *F*^2^ > σ(*F*^2^) is used only for calculating *R*-factors(gt) *etc*. and is not relevant to the choice of reflections for refinement. *R*-factors based on *F*^2^ are statistically about twice as large as those based on *F*, and *R*- factors based on ALL data will be even larger.

### Fractional atomic coordinates and isotropic or equivalent isotropic displacement parameters (Å^2^)

**Table:**

	*x*	*y*	*z*	*U*_iso_*/*U*_eq_	
O1	0.3120 (4)	0.8483 (4)	0.6292 (2)	0.228 (2)	
C14	0.2953 (8)	0.3803 (7)	0.98250 (18)	0.198 (3)	
H14A	0.3992	0.3571	0.9812	0.297*	
H14B	0.2363	0.3039	0.9886	0.297*	
H14C	0.2791	0.4411	1.0111	0.297*	
S1	0.12098 (6)	0.62642 (6)	0.77099 (3)	0.0654 (2)	
C16	0.1285 (2)	0.3775 (2)	0.65770 (8)	0.0549 (5)	
O3	−0.03436 (17)	0.60536 (19)	0.76309 (8)	0.0833 (5)	
C17	0.1732 (3)	0.2664 (2)	0.63569 (9)	0.0576 (6)	
H17	0.2346	0.2163	0.6576	0.069*	
O2	0.1799 (2)	0.75371 (16)	0.76813 (9)	0.0884 (6)	
N1	0.20652 (19)	0.54286 (17)	0.72366 (8)	0.0561 (5)	
C24	0.0421 (3)	0.4723 (3)	0.62915 (11)	0.0734 (7)	
C2	0.4743 (3)	0.4998 (2)	0.73721 (10)	0.0672 (7)	
H2	0.4505	0.4303	0.7594	0.081*	
C1	0.3624 (2)	0.5716 (2)	0.71374 (8)	0.0504 (5)	
C3	0.6222 (3)	0.5311 (3)	0.72779 (13)	0.0800 (8)	
H3	0.6976	0.4824	0.7438	0.096*	
C6	0.3987 (3)	0.6749 (2)	0.68055 (10)	0.0597 (6)	
C18	0.1425 (3)	0.2092 (2)	0.58296 (9)	0.0595 (6)	
C4	0.6589 (3)	0.6330 (3)	0.69519 (11)	0.0744 (7)	
H4	0.7587	0.6534	0.6889	0.089*	
C15	0.1553 (3)	0.4077 (2)	0.71546 (9)	0.0628 (6)	
H15A	0.0636	0.3940	0.7354	0.075*	
H15B	0.2301	0.3486	0.7295	0.075*	
C23	0.2278 (3)	0.1035 (3)	0.56710 (12)	0.0763 (7)	
H23	0.3009	0.0713	0.5901	0.092*	
C5	0.5495 (3)	0.7035 (3)	0.67231 (10)	0.0683 (7)	
H5	0.5751	0.7732	0.6504	0.082*	
C20	0.0073 (5)	0.1869 (3)	0.50109 (13)	0.1054 (11)	
H20	−0.0705	0.2145	0.4791	0.126*	
N2	−0.0240 (4)	0.5520 (3)	0.60771 (12)	0.1084 (9)	
C8	0.1702 (3)	0.5580 (3)	0.83244 (10)	0.0652 (7)	
C10	0.1288 (5)	0.4016 (4)	0.90171 (14)	0.1050 (10)	
H10	0.0720	0.3334	0.9152	0.126*	
C13	0.2949 (3)	0.6014 (4)	0.85973 (15)	0.1027 (11)	
H13	0.3527	0.6688	0.8461	0.123*	
C22	0.2061 (4)	0.0452 (3)	0.51797 (14)	0.0961 (10)	
H22	0.2673	−0.0235	0.5077	0.115*	
C19	0.0291 (4)	0.2479 (3)	0.54918 (12)	0.0904 (9)	
H19	−0.0332	0.3160	0.5592	0.109*	
C25	0.0719 (6)	0.0218 (5)	0.43028 (15)	0.1551 (19)	
H25A	0.0117	0.0767	0.4079	0.233*	
H25B	0.0219	−0.0597	0.4355	0.233*	
H25C	0.1668	0.0071	0.4133	0.233*	
C7	0.2845 (4)	0.7516 (3)	0.65304 (15)	0.1055 (12)	
H7	0.1859	0.7232	0.6543	0.127*	
C21	0.0960 (5)	0.0869 (3)	0.48418 (12)	0.0964 (10)	
C9	0.0865 (3)	0.4586 (3)	0.85373 (12)	0.0816 (8)	
H9	0.0014	0.4297	0.8359	0.098*	
C11	0.2499 (5)	0.4419 (5)	0.92945 (15)	0.1210 (14)	
C12	0.3324 (4)	0.5417 (6)	0.90856 (18)	0.1314 (17)	
H12	0.4158	0.5709	0.9273	0.158*	

### Atomic displacement parameters (Å^2^)

**Table:**

	*U*^11^	*U*^22^	*U*^33^	*U*^12^	*U*^13^	*U*^23^
O1	0.130 (2)	0.192 (3)	0.363 (5)	−0.020 (2)	−0.038 (3)	0.212 (4)
C14	0.248 (7)	0.247 (6)	0.100 (3)	0.073 (6)	−0.044 (4)	0.005 (4)
S1	0.0426 (3)	0.0560 (3)	0.0975 (5)	0.0013 (3)	0.0086 (3)	−0.0120 (3)
C16	0.0484 (11)	0.0527 (12)	0.0636 (13)	−0.0027 (12)	0.0040 (11)	0.0009 (11)
O3	0.0409 (9)	0.0969 (13)	0.1122 (14)	0.0073 (9)	−0.0007 (9)	−0.0069 (12)
C17	0.0535 (13)	0.0545 (14)	0.0647 (14)	−0.0001 (11)	0.0035 (10)	0.0060 (12)
O2	0.0722 (11)	0.0487 (9)	0.1445 (16)	0.0002 (8)	0.0266 (11)	−0.0155 (12)
N1	0.0475 (10)	0.0491 (10)	0.0718 (12)	−0.0060 (8)	0.0032 (9)	−0.0044 (10)
C24	0.0756 (17)	0.0689 (18)	0.0757 (16)	0.0102 (15)	0.0020 (14)	−0.0112 (15)
C2	0.0635 (15)	0.0590 (14)	0.0792 (16)	0.0129 (11)	−0.0054 (13)	0.0094 (13)
C1	0.0453 (11)	0.0465 (11)	0.0595 (12)	0.0036 (9)	0.0008 (10)	−0.0049 (10)
C3	0.0534 (14)	0.0804 (17)	0.106 (2)	0.0212 (14)	−0.0092 (16)	−0.0056 (17)
C6	0.0544 (14)	0.0539 (13)	0.0707 (15)	−0.0020 (10)	−0.0005 (12)	0.0046 (12)
C18	0.0648 (15)	0.0526 (14)	0.0611 (14)	0.0018 (12)	0.0069 (12)	0.0049 (11)
C4	0.0455 (14)	0.0896 (19)	0.0881 (18)	−0.0005 (14)	0.0097 (13)	−0.0230 (17)
C15	0.0679 (15)	0.0540 (13)	0.0666 (14)	−0.0154 (11)	0.0049 (12)	−0.0052 (12)
C23	0.0712 (17)	0.0681 (17)	0.0895 (19)	0.0102 (14)	−0.0001 (14)	−0.0087 (16)
C5	0.0649 (17)	0.0665 (16)	0.0736 (16)	−0.0135 (13)	0.0092 (13)	−0.0028 (13)
C20	0.141 (3)	0.091 (2)	0.085 (2)	0.021 (2)	−0.032 (2)	−0.0105 (19)
N2	0.127 (2)	0.0856 (18)	0.112 (2)	0.0383 (18)	−0.0249 (18)	−0.0046 (16)
C8	0.0452 (12)	0.0717 (17)	0.0787 (16)	0.0009 (12)	0.0043 (11)	−0.0293 (14)
C10	0.134 (3)	0.097 (3)	0.084 (2)	−0.001 (3)	0.008 (2)	−0.0085 (19)
C13	0.0679 (18)	0.142 (3)	0.098 (2)	−0.026 (2)	0.0056 (17)	−0.043 (2)
C22	0.108 (2)	0.082 (2)	0.099 (2)	0.0099 (19)	0.018 (2)	−0.0267 (19)
C19	0.110 (2)	0.0745 (19)	0.086 (2)	0.0275 (18)	−0.0280 (17)	−0.0168 (16)
C25	0.223 (5)	0.151 (4)	0.092 (2)	−0.003 (4)	−0.014 (3)	−0.053 (3)
C7	0.082 (2)	0.084 (2)	0.150 (3)	−0.0029 (17)	−0.018 (2)	0.056 (2)
C21	0.132 (3)	0.086 (2)	0.0709 (18)	−0.006 (2)	0.005 (2)	−0.0170 (17)
C9	0.079 (2)	0.084 (2)	0.0825 (19)	−0.0122 (16)	0.0012 (14)	−0.0170 (17)
C11	0.119 (3)	0.159 (4)	0.085 (3)	0.027 (3)	−0.001 (2)	−0.029 (3)
C12	0.085 (3)	0.216 (5)	0.093 (3)	−0.002 (3)	−0.021 (2)	−0.056 (3)

### Geometric parameters (Å, º)

**Table:**

O1—C7	1.185 (4)	C4—H4	0.9300
C14—C11	1.522 (6)	C15—H15A	0.9700
C14—H14A	0.9600	C15—H15B	0.9700
C14—H14B	0.9600	C23—C22	1.378 (4)
C14—H14C	0.9600	C23—H23	0.9300
S1—O2	1.4148 (18)	C5—H5	0.9300
S1—O3	1.4198 (17)	C20—C21	1.367 (5)
S1—N1	1.649 (2)	C20—C19	1.367 (4)
S1—C8	1.742 (3)	C20—H20	0.9300
C16—C17	1.331 (3)	C8—C9	1.375 (4)
C16—C24	1.434 (4)	C8—C13	1.381 (4)
C16—C15	1.492 (3)	C10—C11	1.351 (5)
C17—C18	1.466 (3)	C10—C9	1.385 (5)
C17—H17	0.9300	C10—H10	0.9300
N1—C1	1.447 (3)	C13—C12	1.404 (6)
N1—C15	1.479 (3)	C13—H13	0.9300
C24—N2	1.145 (3)	C22—C21	1.365 (5)
C2—C1	1.375 (3)	C22—H22	0.9300
C2—C3	1.381 (4)	C19—H19	0.9300
C2—H2	0.9300	C25—C21	1.517 (5)
C1—C6	1.386 (3)	C25—H25A	0.9600
C3—C4	1.367 (4)	C25—H25B	0.9600
C3—H3	0.9300	C25—H25C	0.9600
C6—C5	1.396 (4)	C7—H7	0.9300
C6—C7	1.462 (4)	C9—H9	0.9300
C18—C19	1.377 (4)	C11—C12	1.368 (6)
C18—C23	1.388 (3)	C12—H12	0.9300
C4—C5	1.345 (4)		
			
C11—C14—H14A	109.5	C22—C23—C18	121.2 (3)
C11—C14—H14B	109.5	C22—C23—H23	119.4
H14A—C14—H14B	109.5	C18—C23—H23	119.4
C11—C14—H14C	109.5	C4—C5—C6	121.7 (3)
H14A—C14—H14C	109.5	C4—C5—H5	119.1
H14B—C14—H14C	109.5	C6—C5—H5	119.1
O2—S1—O3	119.94 (12)	C21—C20—C19	122.3 (3)
O2—S1—N1	105.95 (11)	C21—C20—H20	118.9
O3—S1—N1	105.96 (11)	C19—C20—H20	118.9
O2—S1—C8	108.98 (13)	C9—C8—C13	119.4 (3)
O3—S1—C8	107.90 (12)	C9—C8—S1	120.2 (2)
N1—S1—C8	107.49 (10)	C13—C8—S1	120.4 (3)
C17—C16—C24	122.9 (2)	C11—C10—C9	122.0 (4)
C17—C16—C15	121.9 (2)	C11—C10—H10	119.0
C24—C16—C15	115.0 (2)	C9—C10—H10	119.0
C16—C17—C18	131.2 (2)	C8—C13—C12	118.5 (4)
C16—C17—H17	114.4	C8—C13—H13	120.7
C18—C17—H17	114.4	C12—C13—H13	120.7
C1—N1—C15	117.84 (18)	C21—C22—C23	120.8 (3)
C1—N1—S1	117.57 (14)	C21—C22—H22	119.6
C15—N1—S1	116.52 (15)	C23—C22—H22	119.6
N2—C24—C16	177.1 (3)	C20—C19—C18	120.5 (3)
C1—C2—C3	119.9 (2)	C20—C19—H19	119.7
C1—C2—H2	120.0	C18—C19—H19	119.7
C3—C2—H2	120.0	C21—C25—H25A	109.5
C2—C1—C6	119.7 (2)	C21—C25—H25B	109.5
C2—C1—N1	121.3 (2)	H25A—C25—H25B	109.5
C6—C1—N1	118.99 (19)	C21—C25—H25C	109.5
C4—C3—C2	120.7 (2)	H25A—C25—H25C	109.5
C4—C3—H3	119.7	H25B—C25—H25C	109.5
C2—C3—H3	119.7	O1—C7—C6	123.0 (3)
C1—C6—C5	118.4 (2)	O1—C7—H7	118.5
C1—C6—C7	122.1 (2)	C6—C7—H7	118.5
C5—C6—C7	119.5 (3)	C22—C21—C20	117.8 (3)
C19—C18—C23	117.2 (2)	C22—C21—C25	120.6 (4)
C19—C18—C17	124.8 (2)	C20—C21—C25	121.6 (4)
C23—C18—C17	117.9 (2)	C8—C9—C10	120.0 (3)
C5—C4—C3	119.5 (2)	C8—C9—H9	120.0
C5—C4—H4	120.3	C10—C9—H9	120.0
C3—C4—H4	120.3	C10—C11—C12	118.0 (4)
N1—C15—C16	112.3 (2)	C10—C11—C14	122.0 (5)
N1—C15—H15A	109.1	C12—C11—C14	120.0 (5)
C16—C15—H15A	109.1	C11—C12—C13	122.0 (4)
N1—C15—H15B	109.1	C11—C12—H12	119.0
C16—C15—H15B	109.1	C13—C12—H12	119.0
H15A—C15—H15B	107.9		
			
C24—C16—C17—C18	−4.5 (4)	C3—C4—C5—C6	−0.6 (4)
C15—C16—C17—C18	170.8 (2)	C1—C6—C5—C4	0.7 (4)
O2—S1—N1—C1	36.60 (19)	C7—C6—C5—C4	−177.6 (3)
O3—S1—N1—C1	165.03 (17)	O2—S1—C8—C9	156.4 (2)
C8—S1—N1—C1	−79.81 (18)	O3—S1—C8—C9	24.6 (2)
O2—S1—N1—C15	−175.28 (18)	N1—S1—C8—C9	−89.2 (2)
O3—S1—N1—C15	−46.8 (2)	O2—S1—C8—C13	−24.7 (3)
C8—S1—N1—C15	68.32 (19)	O3—S1—C8—C13	−156.5 (2)
C17—C16—C24—N2	−151 (6)	N1—S1—C8—C13	89.7 (2)
C15—C16—C24—N2	33 (7)	C9—C8—C13—C12	0.3 (5)
C3—C2—C1—C6	0.2 (4)	S1—C8—C13—C12	−178.7 (3)
C3—C2—C1—N1	−178.8 (2)	C18—C23—C22—C21	−2.5 (5)
C15—N1—C1—C2	−52.1 (3)	C21—C20—C19—C18	−0.5 (6)
S1—N1—C1—C2	95.6 (2)	C23—C18—C19—C20	−2.7 (5)
C15—N1—C1—C6	128.9 (2)	C17—C18—C19—C20	−178.7 (3)
S1—N1—C1—C6	−83.4 (2)	C1—C6—C7—O1	171.7 (4)
C1—C2—C3—C4	−0.1 (4)	C5—C6—C7—O1	−10.1 (6)
C2—C1—C6—C5	−0.5 (3)	C23—C22—C21—C20	−0.8 (5)
N1—C1—C6—C5	178.5 (2)	C23—C22—C21—C25	−179.6 (4)
C2—C1—C6—C7	177.8 (3)	C19—C20—C21—C22	2.3 (6)
N1—C1—C6—C7	−3.3 (4)	C19—C20—C21—C25	−179.0 (4)
C16—C17—C18—C19	−16.3 (4)	C13—C8—C9—C10	−1.0 (4)
C16—C17—C18—C23	167.7 (2)	S1—C8—C9—C10	177.9 (2)
C2—C3—C4—C5	0.3 (4)	C11—C10—C9—C8	1.2 (5)
C1—N1—C15—C16	−79.8 (3)	C9—C10—C11—C12	−0.5 (6)
S1—N1—C15—C16	132.11 (18)	C9—C10—C11—C14	179.3 (4)
C17—C16—C15—N1	136.8 (2)	C10—C11—C12—C13	−0.3 (6)
C24—C16—C15—N1	−47.5 (3)	C14—C11—C12—C13	179.9 (4)
C19—C18—C23—C22	4.2 (4)	C8—C13—C12—C11	0.4 (6)
C17—C18—C23—C22	−179.5 (3)		

### Hydrogen-bond geometry (Å, º)

**Table:**

*D*—H···*A*	*D*—H	H···*A*	*D*···*A*	*D*—H···*A*
C15—H15*A*···O3	0.97	2.45	2.904 (3)	109
C23—H23···O1^i^	0.93	2.50	3.142 (4)	127

Symmetry code: (i) *x*, *y*−1, *z*.
